# ISG15 in the tumorigenesis and treatment of cancer: An emerging role in malignancies of the digestive system

**DOI:** 10.18632/oncotarget.11911

**Published:** 2016-09-08

**Authors:** Chaohui Zuo, Xinyi Sheng, Min Ma, Man Xia, Linda Ouyang

**Affiliations:** ^1^ Department of Gastroduodenal and Pancreatic Surgery, Translation Medicine Research Center of Liver Cancer, Hunan Cancer Hospital and The Affiliated Cancer Hospital of Xiangya School of Medicine, Central South University, Changsha, Hunan, China; ^2^ Graduate School, University of South China, Hengyang, Hunan, China; ^3^ Laboratory of Digestive Oncology, Hunan Province Cancer Institute, Changsha, Hunan, China

**Keywords:** interferon-stimulated gene 15, interferon, tumorigenesis, digestive system, cancer

## Abstract

The interferon-stimulated gene 15 ubiquitin-like modifier (*ISG15*) encodes an IFN-inducible, ubiquitin-like protein. The ISG15 protein forms conjugates with numerous cellular proteins that are involved in a multitude of cellular functions, including interferon-induced immune responses and the regulation of cellular protein turnover. The expression of ISG15 and ISG15-mediated conjugation has been implicated in a wide range of human tumors and cancer cell lines, but the roles of ISG15 in tumorigenesis and responses to anticancer treatments remain largely unknown. In this review, we discuss the findings of recent studies with regard to the role of ISG15 pathways in cancers of the digestive system.

## INTRODUCTION

Cancers of the digestive system are major public health problems worldwide. Cancers of the stomach, colon, rectum, liver, pancreas, and esophagus are highly prevalent, and rank among the top causes of cancer-related death among men and women in both developed and developing countries [1, 2]. Similar rates of incidence and mortality have been reported for both rural and developed regions of China, the world's most populous nation [3]. As a major route of contact with pathogens, carcinogens, and potentially noxious substances, the need for effective immunity in digestive organs is paramount for protection against infection, malignancy, and inflammation. The destruction of tumor cells by various chemotherapy agents has been shown to induce immunogenic mechanisms [4-6], and considerable efforts have been undertaken in recent years to elucidate mechanisms underlying tumor immunity to identify immune cells and cytokines with antitumor activates with the goal of augmenting chemotherapy and radiation treatments [7, 8].

Induced by viral infection and various cellular stress stimuli, the secretion of interferons (IFNs) by immunomodulatory cells has been shown to stimulate multiple signaling pathways that regulate the induction of the innate immune response [9]. Clinical studies have demonstrated the benefit of IFN-α as an adjuvant to chemotherapy and radiation treatments following the resection of malignant tumors, including cancers of the digestive system [10-13]. Cell culture and animal studies have also shown that treatments that included IFN-α inhibited the proliferation and metastasis of hepatocellular carcinoma [14], gastric cancer, and pancreatic cancer cells [15]. However, recent studies have shown that IFN signaling is also involved in immunosuppression [16]. Therefore, a better understanding of IFN-induced signaling is needed to fully elucidate mechanisms of innate immunity to malignancies.

The IFN-mediated pathways involved in the innate antiviral response include those mediated by IFN-stimulated gene 15 ubiquitin-like modifier (ISG15), Mx GTPase, protein kinase R, and 2′-5′oligoadenylate-synthetase-directed ribonuclease L [17]. The expression of ISG15 is regulated by an IFN-inducible enhancer element at the *ISG15* promoter [18]. The binding of ISG15 to intracellular target proteins requires the sequential action of three enzymes, the expression of which are also induced by IFNs [19, 20]. Perturbations of the ISG15 pathway have been reported in tumors of the bladder [20], ovary [21], prostate [22], breast [23, 24], and oral squamous tissue [25], and the downregulation of ISG15 expression has been shown to be associated with tumor progression [20].

In this review, we describe the biological functions of ISG15, and discuss evidence from recent studies that highlight the potential roles of ISG15 in tumorigenesis and response to treatment in cancers of the digestive system. Limited information is available on the role of ISG15 in digestive system cancers. Therefore, we describe the functions of ISG15 in IFN-mediated immune responses that are relevant to innate immunity to cancer. We limit our discussion of the roles of ISG15 in cancers of nondigestive organs primarily to tumors that arise from epithelial tissues, such as carcinomas of the breast, lung, and urinary bladder, because these are likely relevant to carcinomas of the esophagous, stomach, intestine, colon, liver, and pancreas, which are more common in digestive organs than tumors arising from other tissue types [26].

## IFNS IN TUMOR IMMUNITY

Consisting of a family of secreted pleiotropic cytokines, IFNs function in the induction and regulation of immune responses. Members of the IFN family of cytokines are classified as type 1, −2, or −3 based on their cellular receptors and intracellular signaling mechanisms [27]. Type I IFNs include IFN-α, IFN-β, IFN-ω, IFNε, and IFN-κ in humans [28-31]. Type 1 IFN signaling occurs via binding to IFN-α receptor (IFNAR)-1 and IFNAR2; activation of tyrosine kinase 2 (TYK2) and Janus kinase 1 (JAK1); phosphorylation and heterodimerization of signal transducer and activator of transcription (STAT) 1 and STAT2; and complex formation with IFN regulatory factor 9 (IRF9), forming the IFN stimulated gene factor 3 (ISGF3) complex, which binds to IFN-stimulated response element (ISRE) sequences in IFN stimulated genes (ISGs) [32]. Various studies have also shown that type I IFN-activated TYK2 and JAK1 can also activate STAT4, STAT5, or STAT6, which induce ISGs directly [33, 34], as well as the insulin receptor substrate (IRS) pathways, which induce ISGs through transcription factors yet to be identified [35, 36].

Both IFN-α and IFN-β play key roles in responses to viral infections [37] and in the suppression of tumor cell proliferation and angiogenesis [38]. Treatment with IFN-α has been shown to accelerate tumor necrosis factor (TNF)-induced apoptosis of tumor cells by upregulating Fas expression [39, 40], and to increase the sensitivity of hepatoma cells to chemotherapeutic drugs by inhibiting NF-κB activation [39, 41, 42] or by inhibiting apoptosis mediated by the TNF-related apoptosis-inducing ligand (TNFSF10) [43]. More recently, Zuo et al. [44] reported that the use of transcatheter arterial chemoembolization and IFN-α in combination after curative resection for hepatitis B virus (HBV)-related hepatocellular carcinoma (HCC) improved overall survival and decreased recurrence. However, the mechanism through which such effects are manifested remains unclear.

The only type II IFN in humans, IFN-γ forms a soluble dimer [45] in solution. The expression of IFN-γ is induced primarily in Th1 lymphocytes and natural killer (NK) cells by mitogenic or antigenic stimuli [46] through the activation of Toll-like receptors (TLR) 3, 4, 7 and 9 [47]. Forming a soluble dimer, IFN-γ mediates signaling occurs through the binding of IFN-γ receptor (IFNGR)-1 and IFNGR2; activation of JAK1 and JAK2; and homodimerization or heterodimerization of STAT1 and/or STAT3, forming the gamma-IFN activation factor (GAF), which binds to IFN-γ activated sequences (GAS)-1, GAS2, or GAS3 in ISGs, respectively [48, 49].

Although many studies have shown that IFN-γ functions primarily in innate and adaptive immunity to a range of pathogens, it has also been shown to exhibit cytotoxic activity, inhibit angiogenesis, and increase the expression of major histocompatibility (MHC) class II molecules [50, 51]. In addition, results of studies of colon cancer suggest that IFN-γ may limit tumor progression [52], and enhances the effectiveness of radiotherapy [53]. It has also been shown to induce autophagy and nonapoptotic cell death in HCC via the activation of IFN regulatory factor 1 (IRF1) [54]. Recent cell culture and animal studies have demonstrated that both IFN-α and IFN-γ are involved in the stepwise induction of immunosuppressive dendritic cells (DCs) in melanoma, persistent viral infection, and *Mycobacterium tuberculosis* and human immunodeficiency virus (HIV) infections [16]. However, the mechanism underlying the functional shift in DCs that ultimately resulted in T cell suppression and whether such a mechanism influences tumorigenesis remain unclear.

Type III IFNs include IFN-λ1, IFN-λ2, IFN-λ3, and IFN-λ4, which share structural features with members of the interleukin (IL)-10 cytokine family [55-58]. Type III IFNs require the IFN-lambda receptor 1 (IFNLR1) and IL-10 receptor 2 (IL10RB) to induce signal transduction via type I IFN intracellular pathways [59]. Although type III IFNs stimulate many of the same ISGs as type I IFNS [60], expression of IFNLR1 is restricted to epithelial cells [61], which may limit possible sites of the activation of type III IFN-mediated immunity.

## ISG15 STRUCTURE, EXPRESSION, SECRETION, AND UBIQUITYLATION

**Figure 1:**
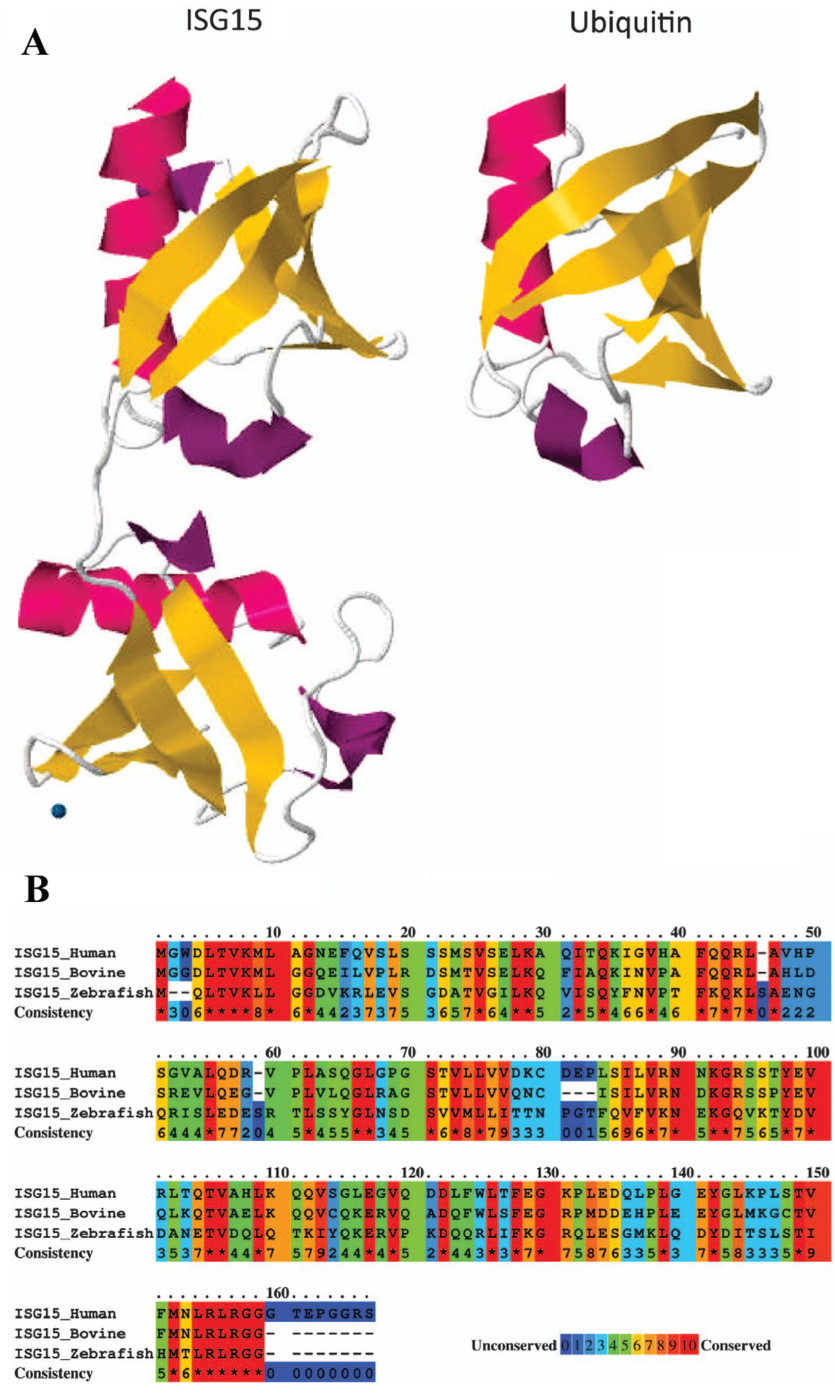
ISG15 protein structure **A.** Comparison of tertiary structures of ISG15 and ubiquitin demonstrating structural homology, as represented by the two ubiquitin domains in ISG15. **B.** Analysis of amino acid sequence similarity between human, bovine, and zebrafish ISG15 orthologs using PRALINE (http://www.ibi.vu.nl/programs/pralinewww/), in which 0 represents least conserved and 10 represents most conserved, as indicated at the alignment position. Amino acid sequence data for ISG15 and ubiquitin were obtained from the Protein Database (http://www.ncbi.nlm.nih.gov/protein/) for 1Z2M and 1UBQ, respectively (figure adapted from Bogunovic et al. [78]).

The ISG15 protein was first described as ubiquitin cross-reactive protein (UCRP) because of cross-reactivity with ubiquitin-specific antibodies [62], and the *ISG15* gene was later annotated as *G1P2* [63]. The molecular structure of the human ISG15 protein has been determined, and orthologs of ISG15 have been identified in mouse, rat, cow, sheep, and fish, but not in yeast, nematodes, or insects, which suggests that it coevolved with IFN signaling pathways in vertebrates [64, 65]. Substantial diversity occurs among ISG15 orthologs, with human ISG15 sharing only 67% and 37% amino acid sequence identity with the bovine and zebrafish orthologs, respectively (Figure 1).

**Figure 2:**
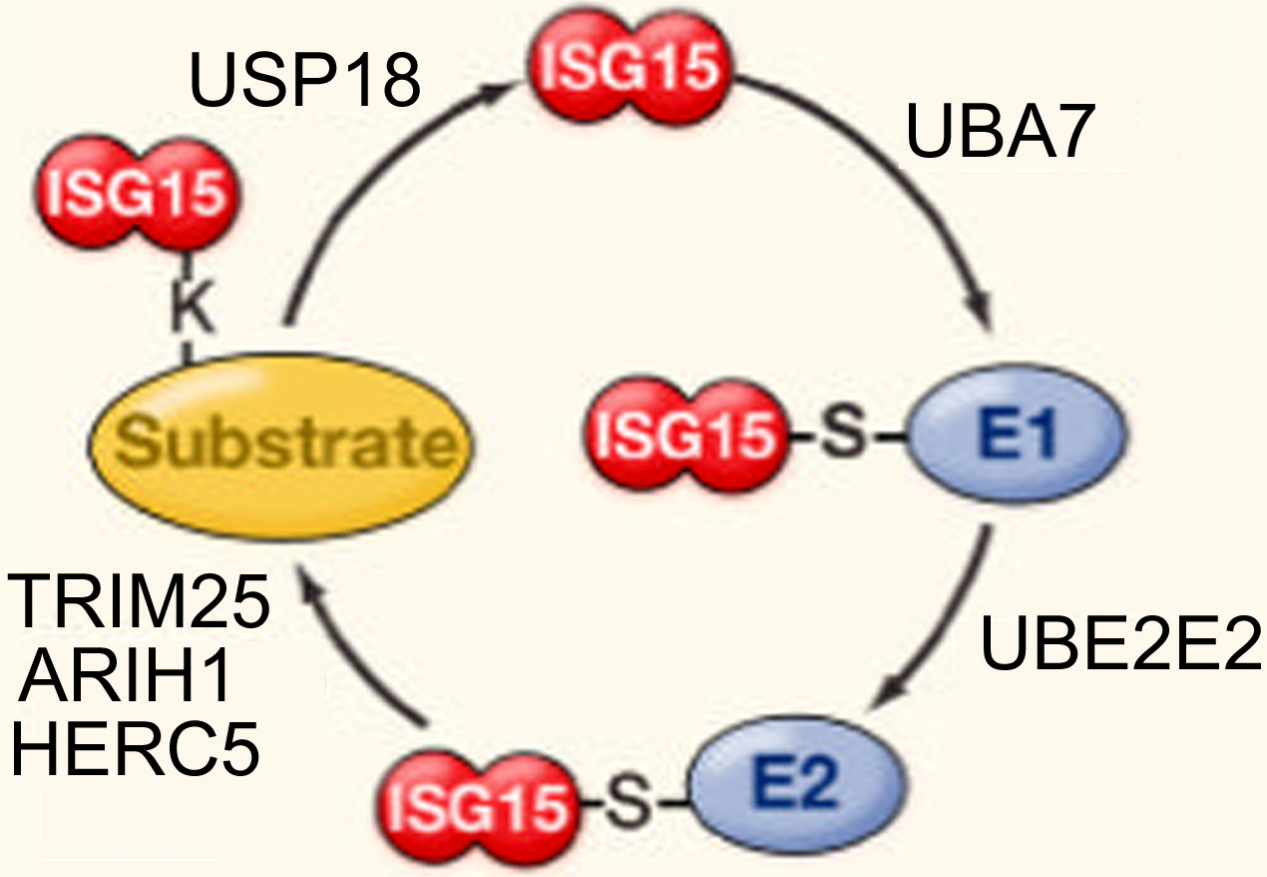
Reactions involved in ISGylation of ISG15 to substrate proteins ATP-dependent activation is catalyzed by UBA7, after which ISG15 is tranferred to UBE2E2. Conjugation to the target protein is catalyzed by TRIM25, ARIH1, or HERC5, and de-ISGylation is catalyzed by USP18 (figure adapted from Skaug and Chen [165]).

All of the ISG15 orthologs contain two tandem domains that have a high level of homology with ubiquitin, through which covalent conjugation with effector proteins occurs by ubiquitylation, also referred to as ISGylation in the case of ISG protein conjugation. Expressed as a 165-aa inactive precursor, eight amino acids are proteolytically cleaved from the carboxyl terminus of the nascent human ISG15 polypeptide to expose an LRLRGG motif that is required for conjugation to lysine-rich ubiquitin binding domains in target effector proteins [66-69]. As depicted in Figure 2, conjugation occurs via a series of three sequential reactions [70] that are similar to that of ubuiquitylation, but occur via separate pathways [71]. The ATP-dependent activation of ISG15 is catalyzed by ubiquitin like modifier activating enzyme 7 (UBA7) [72], by which ISG15 is covalently bound to an E1 carrier protein, and the ubiquitin conjugating enzyme E2 E2 (UBE2E2) [72] catalyzes the transfer of ISG15 to an E2 carrier protein, after which the ligation of ISG15 to the target protein is catalyzed by number of different enzymes, including tripartite motif containing 25 (TRIM25) [73], ariadne RBR E3 ubiquitin protein ligase 1 (ARIH1) [74], and the HECT and RLD domain containing E3 ubiquitin protein ligase 5 (HERC5) [75]. The release of ISG15 from ISG15-target protein conjugate is catalyzed by ubiquitin specific peptidase 18 (USP18) [76].

The expression of ISG15 has been observed in wide range of cell types [65, 77, 78]. In human cells, transcription of ISG15 is induced in response to viral or bacterial pathogens via type I IFN signaling [65, 79]. The effect of IFN-α on ISG15 transcription is mediated by an IFN-inducible enhancer element [18]. Examinations of leukocytes isolated from healthy volunteers have shown that primary cultures of peripheral blood mononuclear cells (PBMCs), T cells (including both CD3^+^CD8^+^ and CD3^+^CD4^+^ cells), granulocytes, neutrophils, fibroblasts, monocytes, macrophages, and monocyte-derived DCs expressed ISG15 in response to IFN-α treatment, and that basal ISG15 expression was detectable in some PBMCs, granulocytes, and monocytes without IFN-α treatment [82].

Cell culture experiments have demonstrated the accumulation of ISG15 protein in the medium of cultured human monocytes, T cells, B cells, and epithelial cell lines treated with IFN-α/β [81, 83], whereas IFN-γ did not induce ISG15 secretion from PBMCs [81]. Both the free and conjugated forms of ISG15 increase in cells exposed to various pathogens and in those treated with type I IFNs [84], and leukocytes isolated from humans with mutant alleles of *ISG15* were shown to secrete ISG in response to bacterial challenge, with a conmittant reduction in intracellular ISG15 protein [80]. However, the *ISG15* gene does not encode an N-terminal secretory sequence. Therefore, ISG15 is likely released from cells via an ER and Golgi independent pathway that is similar to those by which IL-1b and fibroblast growth factor-1 are secreted [85].

Cell culture experiments and bioinformatics analyses have suggested that ISG15 is involved in mitotic processes, including cell cycle arrest [86], but substantial experimental evidence of the role of ISG15 in mitotic processes is currently lacking. A recent study by Fan et al. showed that ISG15 can modify ubiquitin, which fixes ubiquitin to its substrate, forming ISG15-ubiquitin substrate complexes [87]. These ISG15-ubiquitin-substrate complexes are resistant to proteasome-mediated degradation, thereby negatively regulating cellular protein turnover. However, whether ISG15-ubiquitin conjugation is involved in mitotic processes is unknown. Future investigations of the role of ISG15-ubiquitin conjugation in cell cycle arrest are required to determine whether ISG15-ubiquitin-substrate complex formation might contribute to tumorigenic processes.

## ISG15 IN INNATE IMMUNITY

The extracellular and intracellular immunomodulatory activities of ISG15 differ distinctly. Secreted ISG15 stimulates the production of IFN-γ from lymphocytes and the proliferation of NK cells [69], whereas ISGylation of intracellular ISG15 protects ISG15-conjugated proteins from degradation via the ubiquitin/26S proteasome pathway [88, 89], a mechanism which has been shown to stabilize the ISG15-IRF3 conjugate by inhibiting the ubiquitylation of IRF3 via peptidylprolyl cis/trans isomerase, NIMA-interacting 1 (PIN1) [90, 91]. In cells, more than 300 substrate proteins have been identified as potential targets of ISGylation, which includes proteins involved in a diverse range of cellular processes, such as cytoskeleton structure, RNA splicing, translation, stress response, and chromatin remodeling/polymerase II transcription [84].

## ISG15 IN IMMUNITY TO PATHOGENS

The expression of ISG is strongly induced by viral infection, primarily via type I IFN signaling [79, 92]. Both conjugated and free ISG15 exhibit antiviral activities against a wide range of viruses [84, 93], including influenza virus, vaccinia virus, vesicular stomatitis virus, Sendai virus, Japanese encephalitis virus, Newcastle disease virus, avian sarcoma leucosis virus, human papilloma virus (HPV), HIV-1, Ebola virus-like particles, dengue virus, and west Nile virus [94]. Evidence indicates that ISG15 targets newly synthesized proteins, and experiments using cultured cells as a model for HPV infection showed that ISGylation of the L1 capsid protein of HPV inhibited viral replication [84]. However, the inhibition of viral replication by ISG15 has been shown to be cell-type or species specific for at least some viruses [93], and ISG15 was reported to exhibit proviral effects in a cell culture model of chronic HCV infection [95, 96]. Therefore, the regulatory effects of ISG15 on the innate response might vary under certain circumstances.

Studies of *ISG15*−/− mice have shown that the ability of ISG15 to inhibit the replication of the influenza B virus was dependent on ISGylation. Clinical studies have shown that ISG15-deficient patients exhibit cellular, immunological, and clinical signs of enhanced IFN-α/β immunity, and that the ISG15 is a nonredundant stimulator of IFN-γ production [97]. While these patients did not exhibit an increased rate of severe viral disease, they exhibited increased susceptibility to tuberculosis and decreased IFN-γ production, and their cells were depleted of USP18, which served to downregulate type I IFN signaling by blocking the recycling of ISG15 [97]. These findings show that ISG15 might also function as a negative regulator of type I IFN-mediated immunity.

## ISG15 IN TUMOR IMMUNITY

The activation of type I IFN signaling pathways is a key component of innate immunity to cancer [27]. Cell culture experiments have shown that IFN-α, IFN-β, and IFN-γ act directly on cells to induce caspase-mediated apoptosis in a variety of tumor cell types [38, 98, 99]. The suppressive effects of type I IFNs on tumor growth have led to investigations of the use of IFN-α/−β for augmenting the treatment of various cancers, including colon cancer, HCC, leukemia, and melanoma [11, 12, 44, 100-103], with some benefits reported. The expression of ISG15 has been implicated in the regulation of type I IFN expression and the activation of NK cells, both of which are important mediators of tumor immunity [104]. The secretion of ISG15 by melanoma cells has been shown to modulate the phenotype of tumor-infiltrating DCs by inducing the expression of cadherin 1 (CDH1) [105], which has been shown to reduce the migratory behavior of DCs *in vitro* [106].

In a recently published study, Cunningham et al. reported the stepwise induction of immunosuppressive DCs through sequential type II IFN and type I IFN signaling in a mouse xenograft model of melanoma [16], in which IFN-γ induced the de novo development of naive DCs that subsequently exhibited T cell suppressive properties following type I IFN activation. This mechanism was also shown to be involved in persistent lymphocytic choriomeningitis virus, HIV, and *M. tuberculosis* infections [16]. Although both viruses and cancer can induce the secretion of type I IFNs, whether patterns of enhanced ISG15 expression and ISGylation in cancer cells represent cellular responses to cancer or are merely by-products of cellular processes that have been altered by tumorigenesis is unclear. Future studies of stage-specific changes in type I IFN expression, ISG15 expression, and ISGylation are needed to further clarify the role of ISG15 in innate tumor immunity.

## ISG15 IN TUMORIGENESIS AND TREATMENT OF NONDIGESTIVE SYSTEM CANCERS

Cell culture experiments have shown that the levels of ISG15 expression and ISG15-conjugated proteins in human breast cancer, prostate cancer, ovarian cancer, and melanoma cell lines were higher than those in normal human fibroblasts [107]. Elevated ISG15 expression and ISG15-conjugates have also been reported in a variety of human tumors, including melanoma [105], oral squamous cell carcinoma [108, 109], and malignancies of the breast [107, 110], endometrium [107], and bladder [111]. Investigations of cancers of the digestive system might benefit from information obtained in studies of tumors originating of other types of epithelial tissues in nondigestive organs, due to functional and structural similarities at the tissue level.

## ISG15 IN BREAST CANCER

Desai et al. [107] reported that the intracellular levels of ISG15 expression and ISG15-conjugates in primary tumor cells from breast or endometrium cancer patients were higher than those in healthy breast and endometrium tissues. In tissue culture experiments, the level of ISG15 expression in breast cancer cell lines was inversely proportional to the level of polyubiquitylated proteins (non-ISG ubiquitylation), and the siRNA-mediated knockdown of ISG15 increased the level of polyubiquitylation [107]. These data suggested that ISG15 expression is upregulated in at least some cancers, and that the overexpression of ISG15 in tumor cells interferes with cellular protein turnover by inhibiting polyubiquitylation.

Acinar formation is a characteristic morphological feature of breast ductal cells, which is typically absent in ductal cell carcinoma. Desai et al. also showed that ISG15 and UBE2E2 (formerly UBCH8) disrupt acinar formation in ductal cell carcinoma by inhibiting F-actin polymerization, as evidenced by the formation of acini by cultured ZR-75-1 breast carcinoma cells in which ISG15 expression was knocked down by short hairpin ISG15 RNA [23]. The knockdown of ISG15 expression also reduced ZR-75-1 cell motility in cell migration assays, compared to that of ZR-75-1 cells in which ISG15 was not knocked down [23]. Jeon et al. [112] reported that ISG15 functioned as a tumor suppressor in a mouse model of breast cancer by demonstrating that it promoted anchorage-dependent cell growth in response to doxorubicin treatment. As Desai et al. pointed out, reports of constitutive ISG15 expression in other cancer cell lines suggest that the effects of ISG15-mediated ISGylation on the cytoskeleton architecture in breast cancer cells [24] might also be associated with tumorigenesis in cancers originating from other tissue types.

In a recent study of breast cancer, Burks et al. [113] found that extracellular free ISG15 increased the number of infiltrating NK cells in xenografted tumors in mice, which suppressed tumor growth, and cell culture experiments showed that intracellular free ISG15 enhanced MHC class I expression by breast cancer cells. Although their findings suggest that free ISG15 might be useful for augmenting treatments for breast cancer, how free ISG15 stimulated MHC class I expression in the xenografted cells was not entirely clear. Future studies of the immunomodulatory effects of free intracellular ISG15 in breast cancer cells might benefit from an in-depth examination of its effects on ISG15-mediated protein conjugation.

## ISG15 IN URINARY BLADDER CANCER

In one study of human urinary bladder cancer, upregulated ISG15 expression was detected in bladder tumor cells, compared with the level of ISG15 expression in healthy uroepithelium, and a positive correlation was observed between tumor progression and the stage-specific upregulation of ISG15 expression [111]. Unlike the findings of studies of breast cancer, only free ISG15 was detected in bladder cancer tumors by western blotting, and immunohistochemistry showed that the majority of free ISG15 in tumor cells was located in the nucleus. Although these results suggest that ISG15 might function as an oncoprotein in bladder cells under certain circumstances, the underlying malignancy-causing mechanism appeared to differ from the ISGylation-related mechanism observed in breast cancer, which may be related to differences between the structure and function of bladder epithelium versus those of the glandular tissue of the breast.

## ISG15 IN LUNG CANCER

Isotypes of p63 containing the N-terminal transactivation domain, which normally induce cell cycle arrest and apoptosis, are suppressed in cancer cells exhibiting elevated expression of ΔNp63α, which contains a C-terminal transactivation-inhibitory domain. In the non-small-cell human lung carcinoma cell line, H1299, Jeon et al. showed that doxirubicin treatment reduces tumor cell proliferation via UBA7-mediated cleavage of ΔNp63α, and that ISG15 depletion inhibited the antitumor effects of doxirubicin [112]. Although UBA7 is detectable in healthy jejunum, colon, liver, and lung tissues, McLaughlin et al. found that UBA7 was not detectable in 14 human lung cancer cell lines [114]. In contrast to the role of ISG15 in breast and urinary bladder cancer, these findings suggest that ISGylation can suppress tumorigenesis. However, in K-rasG12D and p53 deficient mouse models of lung cancer, no significant difference in tumor progression was observed between UBA7^−/−^ and UBA7^+/+^ mice in either model [115, 116]. Future studies of lung cancer should seek to further elucidate the role of ISGylation in doxirubicin-induced tumor suppression.

## TUMOR-INDUCED CHANGES IN ISG15 EXPRESSION IN NONDIGESTIVE SYSTEM CANCERS

In contrast to the abovementioned reports of enhanced ISG15 expression and ISGylation in cancer cell lines and tumor cells, expression profiling and cytogenetic analyses have reported reduced expression of ISGs in immune cells [117], myeloid leukemia cells [118, 119], and cells from colorectal [120, 121], breast [122], prostate [123], and melanoma [124] tumors. In addition, cell culture studies have implicated epigenetic silencing by the DNA methylation of IFN-responsive genes, including *STAT1*, TNF receptor superfamily member 10a (*TNFRSF10A*, formerly *TRAIL-R1*), *IRF-7*, and death associated protein kinase (*DAPK*), in the downregulation of IFN signaling in cervical cancer [125], Li-Fraumeni syndrome [126], colon cancer [127], renal carcinoma [128], melanoma [128], and lymphocytic leukemia [129]. These results indicate that future investigations of ISG15 as a biomarker of cancer should consider tumorigenesis-induced changes in the regulation of ISG expression.

Cell culture experiments have shown that monocyte-derived DCs can be activated by secreted ISG15 isolated from the media of melanoma cell lines[105], and that the addition of anti-ISG15 antibodies to the medium inhibited the expression of CDH1, a marker of DC activation. Given that melanoma is a highly metastatic cancer, these results suggest that melanoma-secreted ISG15 may have contributed to the recent findings of Cunningham et al. [16] regarding the generation of immunosuppressive DCs in melanoma, perhaps through the ISG15-mediated induction of type I IFN signaling pathways. However, future studies are needed to determine the effects of melanoma-secreted ISG15 on local DC migration and T cell cytotoxicity to determine whether secreted ISG15 contributes to melanoma progression and metastasis.

## ISG15 IN CANCER OF THE DIGESTIVE SYSTEM

The prognosis for patients with a malignancy of the digestive system is generally poor [130, 131], especially for patients with liver or pancreatic cancer [98, 132, 133] because they are most often diagnosed during the advanced stages of disease [133, 134]. Studies of cancers of the digestive system have identified specific perturbations of the ISG15 pathway, including the dysregulation of ISG15 expression, sequence variation of ISG15 pathway genes, and ISGylation [107, 135, 136], including elevated ISG15 expression in tumors of the esophagus [139], stomach [63], colon/rectum [140], liver [136], and pancreas [141]. A list of the studies reviewed herein that are directly relevant to the role of ISG15 in digestive system cancer is provided in Table 1.

**Table 1:** Studies directly relevant to the role of ISG15 in cancers of the digestive system

Authors and year published	Location /cell culture	Digestive Organ
Matsumura et al. 2005 [161]	Cell culture	Esophageal cancer
Chen et al. 2009 [137]	China	Esophageal cancer
Yan et al. 2012 [138]	China	Esophageal cancer
Tao et al. 2015 [139]	China	Esophageal cancer
Jinawath et al. 2004 [63]	Japan	Gastric cancer
Yang et al. 2007 [142]	Korea	Gastric cancer
Shen et al. 2013 [135]	China	Gastric cancer
Karpf et al. 1999 [127]	Cell culture	Colorectal cancer
Zimmer & Thomas 2002 [120]	Cell culture	Colorectal cancer
Cabrera et al. 2003 [121]	Spain	Colorectal cancer
Desai et al. 2006 [107]	USA	Colorectal cancer
Talvinen et al. 2006 [140]	Finland	Colorectal cancer
Lee et al. 2010 [148]	Korea	Colorectal cancer
Lee et al. 2012 [143]	Cell culture	Colorectal cancer
Kim et al. 2014 [131]	Korea	Colorectal cancer
Roulois et al. 2015 [164]	Cell culture	Colorectal cancrer
Fan et al. 2011 [132]	China	Liver cancer
Wan et al. 2013 [156]	Cell culture	Liver cancer
Konishi et al. 2013 [155]	Japan	Liver cancer
Li et al. 2014 [136]	China	Liver cancer
Hou et al. 2014 [153]	China	Liver cancer
Qiu et al. 2015 [154]	China	Liver cancer
Künzli et al. 2002 [144]	Cell culture	Pancreatic cancer
Ina et al. 2010 [141]	Cell culture	Pancreatic cancer
Sainz et al. 2014 [157]	Spain	Pancreatic cancer

## ISG15 IN ESOPHAGEAL CANCER

Based on a membrane array analysis of tumor tissues from patients with squamous cell (ESCC), Chen et al. [137] found that ISG15 expression was associated with ESCC. In addition, they found that the ubiquitin conjugating enzyme E2 S (UBE2S, formerly E2-EPF) was a significant predictor of tumor burden, whereas ISG15 was not. Based on a microarray analysis of tumor tissues, Yan et al. [138] found that ISG15 expression was upregulated in ESCC, and the expression profile and pathways analyses showed that ISG15 expression was associated with ESCC tumorigenesis. In a more recent study, Tao et al. [139] reported that ISG15 expression was higher in ESCC tumors than in matched control tissues. Their analysis showed that ISG15 mRNA expression was an independent prognosticator of ESCC, and that ISG15 expression was significantly associated with clinical outcome in patients with a history of ethanol consumption, whereas no significant association was observed for patients without a history of drinking alcohol [139]. The findings of these studies suggest that ISG15 expression is a useful prognostic biomarker in ESCC patients, and that future investigations of predictors of ESCC outcome might consider both ISG15 and UBE2S.

## ISG15 IN GASTRIC CANCER

In a microarray analysis of gene expression in tumor samples from patients with diffuse-type gastric cancer, Jinawath et al. [63] reported that ISG15 (formerly G1P2) expression was upregulated in more than 50% of the tumors examined, compared with that in noncancerous gastric mucosa, ranking as the 32nd most highly expressed transcript among the differentially expressed genes identified. Later microarray studies by Yang et al. [142] and Shen et al. [135] also identified increased ISG15 expression in gastric cancer, relative to that in healthy gastric epithelium. Based on the previous report of ISG15-induced CDH1 expression on DCs [105], Jinawath et al. speculated that ISG15 expression might allow diffuse-type gastric tumor cells to evade the innate antitumor immune response by suppressing antigen presentation by DCs. Future studies of gastric cancer should investigate whether the increased expression of ISG15 contributes to tumor progression through an immunosuppressive mechanism similar to that described in the recent report by Cunningham et al. of IFN-induced immunosuppression of DCs in melanoma.

## ISG15 IN COLORECTAL CANCER

Similar to their findings of increased ISG15 expression and ISGylation in breast and endometrium tumors, Desai et al. [107] also found that the levels of ISG15 expression and ISG15-conjugated proteins were elevated in tumors from two colon cancer patients, compared with the levels of each in healthy colon tissues. Lee et al. [143] examined the effects of constitutive expression of the hematopoietic IFN-inducible nuclear protein, Absent in Melanoma 2 (AIM2), on gene expression in the high-level microsatellite unstable colorectal cancer cell line, HCT116. They identified ISG15 among the 111 transcripts that were upregulated by AIM2, compared with expression levels in AIM2-negative HCT116 cells, and reported a positive correlation between the expression of ISG15 and AIM2 in ten different IFN-γ-treated colorectal cancer cell lines [143]. However, the mechanism by which AIM2 stimulates ISG15 expression in the absence of type I IFNs and the downstream effects of AIM2-induced ISG15 expression and ISGylation in colon cancer cells are unclear.

Elevated expression of the tumor-associated glycoprotein, galectin 3 binding protein (LGALS3BP, formerly 90K and MAC-2-BP), has been reported in various types of cancer, including pancreatic [144], gastric [145], and colon cancer [146], and the expression of LGALS3BP has been shown to be associated with poor prognosis in colon cancer [146, 147]. Secreted LGALS3BP can inhibit colon cancer progression through binding to CD9/CD82, which suppresses Wnt/beta-catenin signaling via an ISG15-dependent proteasomal-ubiquitination mechanism [148]. However, LGALS3BP expression is downregulated in advanced colorectal cancer. Future studies are needed to investigate the role of ISG15-dependent Wnt/beta-catenin signaling suppression in cancers of the digestive system.

The penta-span transmembrane glycoprotein, prominin 1 (PROM1, formerly CD133), has been used as a biomarker for the identification and isolation of cancer stem cells (CSCs) from various malignancies, including colorectal cancer [149-151]. Based on their analysis of PROM1 expression in a mouse xenograft model of colorectal cancer, Shmelkov et al. [152] suggested that PROM1-positive colon cancer cells may differentiate into more aggressive PROM1-negative cells during tumorigenesis and metastasis. In a transcriptome analysis of colorectal cancer, Kim et al. [131] found that the level of expression was higher in PROM1-positive cells, compared with that in PROM1-negative colorectal cancer cells. The findings of these studies collectively suggest that ISG15 expression may be involved in the PROM1-positive to PROM1-negative transition of CSCs in colorectal tumors.

## ISG15 IN LIVER CANCER

In a recent study by Li et al [136], the expression of ISG15 was found to be associated with tumor grade, metastasis, and survival in HCC patients, and ISG15 was shown to promote tumor cell proliferation and migration by blocking the interaction between X-linked inhibitor of apoptosis (XIAP) and the apoptosis inhibitor, baculoviral IAP repeat containing 5 (BIRC5, formerly survivin). The siRNA-mediated knockdown of ISG15 expression inhibited tumor progression and increased survival in mice with xenograft tumors, demonstrating that ISG15 promotes HCC tumorigenesis and metastasis. In another recent study, Hou et al. [153] found that the level of the ISG, DEXD/H-box helicase 58 (DDX58, formerly RIG-I), was significantly lower in human HCC tumor cells than that in noncancerous hepatocytes, and that patients with low DDX58 expression had shorter survival and poorer responses to IFN-α therapy.

Both ISG15 and DDX58 are downstream effectors in IFN-α signaling, and DDX58 has been shown to enhance IFN-α-induced ISG15 expression in HBV-related HCC. [154] Future studies of HCC might benefit from investigating the underlying cause of the difference in the expression levels of ISG15 and DDX58 in an effort to determine whether tumor-induced changes in IFN-α-induced signaling contribute to tumorigenesis. In a study of HCV-induced HCC, Konishi et al. [155] found that single nucleotide polymorphisms (SNPs) of *IFN-3* (formerly *IL-28B*) were associated with ISG15 expression in HCV-induced HCC tumors in humans, which indicates that future investigations of ISG15 as a prognostic biomarker in HCC patients should examine the *IFN-λ3* genotype of patients to consider possible genetic influences on the correlation between ISG15 and HCC.

The downstream effects of type I IFN-induced ISG expression in nonviral-induced HCC may differ from that in HBV- or HCV-induced HCC tumors. Wan et al. [156] demonstrated that, although IFN-α induced ISG15 expression in the human HCC cell line, HepG2, IFN-α treatment did not induce apoptosis, whereas the transient overexpression of ISG15 induced apoptosis in HepG2 cells. They also found that transient ISG15 overexpression increased ISG15-mediated ubiquitylation in HepG2, whereas IFN-α treatment did not. These results suggested that ISGylation might be altered in some HCC tumors. Future studies of nonviral-induced HCC should examine a possible link between reduced ISGylation and malignant HCC phenotype.

## ISG15 IN PANCREATIC CANCER

Ina et al. [141] performed a microarray analysis of gemcitabine resistance in 11 cell lines. They found that upregulated gemcitabine resistance in cultured pancreatic cancer cells, and that the siRNA-mediated knockdown of ISG15 expression restored gemcitabine sensitivity. Sainz et al. [157] showed that ISG15 secretion by tumor-associated macrophages (TAMs) stimulated the development of CSCs in a mouse model of pancreatic ductal carcinoma, and that secretion of IFN-β by CSCs maintained the secretion of by TAMs, thereby stimulating the continued development of CSCs. Future studies should investigate whether the contribution of ISG15 to the development and maintenance of CSCs is involved in chemotherapy resistance in pancreatic cancer.

## ISG15 IN RESPONSES TO ANTICANCER THERAPIES

Various chemotherapeutic agents have been shown to increase, and ISG15 expression has been shown to be associated with tumor response to IFN-α treatment, chemotherapy, and radiotherapy [158, 159], as well as the survival of patients with acute promyelocytic leukemia, ovarian cancer, or breast cancer [21, 110]. Therefore, identifying the that mediate the chemosensitivity of tumor cells may help identify potential therapeutic targets for overcoming chemoresistance and improving survival.

Camptothecin has been shown to induce the expression of ISG15 in human fibrosarcoma cells [160], which suggests that ISG15 can function as a tumor suppressor. Matsumura et al. [161] found that 5-fluorouracil (5-FU) stimulated IFN-mediated signaling pathways in three human ESCC cell lines, which included the upregulation of ISG15 in both the presence and absence of type I IFNs, and reported that IFN-α/β augmented the suppressive effects of 5-FU on ESCC cell growth. Two studies of tumor tissues from gastric cancer patients found that the level of ISG15 mRNA expression was significantly higher in irinotecan-sensitive tumors than that in the irinotecan-resistant tumors [135, 162]. However, neither study identified ISG15 expression as a prognosticator of irinotecan response, and Ina et al. reported that upregulated gemcitabine resistance in pancreatic cancer cells [141]. The findings of these studies collectively suggest that the contribution of ISG15 to tumor chemosensitivity may be indirect and tissue specific.

As mentioned in a previous section above, Jeon et al. [112] found that chemosensitivity to doxorubicin in cultured human breast cancer cells was dependent on ISG15-mediated conjugation of an alternative splice variant, ΔNp63α, of tumor protein 53 (TP53, formerly p53). Variable expression of ΔNp63α was observed in doxorubicin resistant cells, which was found to suppress the doxorubicin-induced proapoptotic transactivity of other TP53 family proteins, and promoted anchorage independent growth and tumor sphere formation in a mouse xenograft model. In doxorubicin-sensitive cells, ISGylation of ΔNp63α by ISG15 restored TP53 activity and caspase-2 (CASP2) activation, and blocked anchorage independent growth and tumor formation in the presence of doxorubicin [112]. These findings reveal a mechanism by which can function as a tumor suppressor, and suggest that screening tumors for ΔNp63α expression might be beneficial for selecting the optimal treatment strategy for breast cancer patients [163]. These findings also highlight the need for future investigations of the role of ISG15-mediated conjugation in tumorigenesis and chemosensitivity in other types of cancer.

In a recent cell culture study, Roulois et al. [164] showed that ISG15 expression was highly upregulated in LIM1215 colorectal cancer cells treated with the DNA methylation inhibitor, 5-aza-2-deoxycytidine (5-AZA-CdR). The addition of JAK1/JAK2 or JAK3 inhibitors to the 5-AZA-CdR-treated cells abolished ISG15 expression, and the shRNA-mediated knockdown of DDX58 partially reduced ISG15 expression in the 5-AZA-CdR-treated LIM1215 cells, indicating that 5-AZA-CdR-induced ISG15 expression is induced via type I IFN signaling pathways. These findings support a tumor suppressor role for ISG15 in colorectal cancer, and suggest that DNA methylation might contribute to the alterations in ISG15 expression and ISG15-mediated ISGylation that have been widely reported in various types of cancer.

## CONCLUSIONS

Whether ISG15 functions as a tumor suppressor or promotes tumorigenesis remains largely unclear, due at least in part to the multitude of cellular process in which ISG15-conjugates are involved. With regard to cancers of the digestive system, the bulk of the most compelling current evidence suggests that ISG15 can function as a tumor suppressor, based on correlations of enhanced ISG15 expression and ISGylation with responses to various chemotherapeutic drugs. Future studies that provide a better understanding of the function of ISG15 in healthy, noncancerous cells will be indispensible with regard to determining whether ISG pathways are merely altered by tumorigenic processes, or are usurped to serve oncogenic functions during tumorigenesis, resulting in neoplasia.

The latter case implies that ISGs with strong apoptotic potential are inactivated during tumorigenesis, but there is no clear evidence that ISG15 or ISG15-conjugated proteins meet such a description. Evidence of the role of ISG15-mediated conjugation of cytoskeletal proteins in breast cancer could explain the altered morphology of tumor cells, but it is unclear how cell division might be affected by ISGylation. Future studies that provide a better understanding of the interaction between the ubiquitin and ISG15 conjugation pathways are needed to determine how the effects of tumorigenesis on one might influence processes mediated by the other. Determining whether ISG15-ubiquitin-substrate complex formation plays a role in tumorigenesis is an important research objective.

Demonstrating both cytokine- and chemokine-like properties when secreted by melanomas, secreted ISG15 might be considered a more practical therapeutic target for augmenting currently available anticancer treatment strategies. Given the high incidence, suboptimal therapeutic response, and poor prognosis of patients with cancers of the digestive system, elucidating the effects of secreted ISG15 on local tumor immunity should also be considered an important objective for future research.
